# The role of cellular crosstalk in the progression of diabetic nephropathy

**DOI:** 10.3389/fendo.2023.1173933

**Published:** 2023-07-17

**Authors:** Keying Zhang, Zhangning Fu, Yifan Zhang, Xiangmei Chen, Guangyan Cai, Quan Hong

**Affiliations:** National Clinical Research Center for Kidney Diseases, State Key Laboratory of Kidney Diseases, Beijing Key Laboratory of Kidney Disease Research, First Medical Center of Chinese PLA General Hospital, Nephrology Institute of the Chinese People’s Liberation Army, Beijing, China

**Keywords:** crosstalk, diabetic kidney disease, diabetic nephropathy, intercellular communication, cellular crosstalk

## Abstract

Diabetic nephropathy (DN) is one of the most common complications of diabetes, and its main manifestations are progressive proteinuria and abnormal renal function, which eventually develops end stage renal disease (ESRD). The pathogenesis of DN is complex and involves many signaling pathways and molecules, including metabolic disorders, genetic factors, oxidative stress, inflammation, and microcirculatory abnormalities strategies. With the development of medical experimental techniques, such as single-cell transcriptome sequencing and single-cell proteomics, the pathological alterations caused by kidney cell interactions have attracted more and more attention. Here, we reviewed the characteristics and related mechanisms of crosstalk among kidney cells podocytes, endothelial cells, mesangial cells, pericytes, and immune cells during the development and progression of DN and highlighted its potential therapeutic effects

## 1 Introduction

Diabetic nephropathy (DN) is one of the most frequent and serious microvascular complications of diabetes (1, 2). According to the World Health Organization, the worldwide prevalence of diabetes was estimated to rise from 2.8% in 2000 to 4.4% in 2030. The number of people with diabetes would rise from 171 million to 366 million during the three decades (3). It has been estimated that more than 40% of patients with diabetes would develop chronic kidney disease (CKD), and eventually develop end stage renal disease (ESRD) (4).

Mitochondrial dysfunction leads to microvascular dysfunction (5). Subsequently, the crosstalk with mesangial cells and podocytes further promotes the development of DN (6). Greka et al. also found that there is a close relationship between the decrease in the number of podocytes and the changes in podocyte shape (mainly manifested as podocyte foot process effacement). Podocytes surrounding the glomerular vascular wall, provide an anatomical location for crosstalk between podocytes and endothelial cells. Studies have also found that endothelial cells and podocytes of the filtration barrier can directly interact with Glomerular Basement Membrane (GBM) in a hyperglycemic environment, or regulate with mesangial cells through crosstalk to promote the progression of DN (6).

Renal tubulointerstitial lesions also play a very important role in the development of DN, for their ability to predict the prognosis of renal disease (7). Renal tubulointerstitial injury appears in the early stage of DN and precedes glomerular lesions (8). Interstitial fibrosis in DN is closely related to the injury of renal tubular epithelial cells. Tubular epithelial cells (TECs), the most important cellular component of the renal interstitium, have the function of reabsorption and excretion. Targeted inhibition of renal tubule SGLT2 protects renal function in patients with DN (9). The hyperglycemic and hypoxic environment of DN promotes renal interstitial vascular endothelial cells and pericytes to differentiate into fibroblasts. The dysfunction of endothelial cells aggravates renal tubular epithelial cell apoptosis and tubular atrophy. Damaged renal tubular epithelial cells further drive inflammatory cells into the tubulointerstitial. Moreover, renal tubular cells can interact with pericytes and infiltrating inflammatory cells, participating in the progression of DN (10–12).

Therefore, the crosstalk among kidney cells and interaction with immune cells play an important role in the occurrence and progression of DN. Many reports have confirmed the molecular mode of crosstalk, but there is a lack of systematic understanding. This paper summarizes the molecular mechanism of the interaction among different cells, hoping to provide new ideas for further research on the pathogenesis of DN and drug development.

## 2 Materials and methods

A systematized narrative review was performed to identify the crosstalk of DKD. This review uses the literature study. The PubMed was searched to relevant articles. The keywords are crosstalk, diabetic nephropathy, intercellular communication, cellular crosstalk. A filter was placed to include only articles’ IF≥5. In addition, the reference lists of eligible studies were hand searched.

The inclusion criteria were:

1. Primary literature. The references must be authentic, complete, easy to search and verify, and avoid wrong citations.
2. The article focuses on the study of diabetic nephropathy caused by type 2 diabetes mellitus, especially the molecular mechanisms among renal cells.
3. The references mainly concentrate on basic research and pathogenesis of diabetic nephropathy.

The exclusion criteria were:

1. Secondary literature.
2. The references only focus on the drug research and treatment of diabetic nephropathy without considering the underlying mechanism.
3. Only the articles of single gene sequencing or simple experimental design were completed.

## 3 Result

### 3.1 Endothelial cell and podocyte

Podocytes and endothelial cells maintain capillary homeostasis by crosstalk in the normal physiological process. In the pathological condition of hyperglycemia, hemodynamic abnormality promotes the injury of podocytes and endothelial cells. The activation of intracellular signaling molecules leads to the secretion dysregulation of vascular growth factors such as vascular endothelial growth factor (VEGF) and TGF-β, which are involved in the regulation of abnormal angiogenesis, and eventually albuminuria (13).

#### 3.1.1 Vascular endothelial growth factor: VEGF-A, B, C

The VEGF family mainly consists of three members. They are polypeptides that belong to the cystine-knot super-family of signaling proteins. A variety of subtypes are formed due to different exon shears, which constitute the vascular endothelial growth factor subtype family. Among them, VEGF-A plays a major role in vasculogenesis (14), and is therefore considered as the angiogenic factor. VEGF-A overexpression can cause glomerulomegaly, mesangial proliferation, podocyte effacement and lead to albuminuria (15). VEGF-B is a crucial factor in promoting abnormal lipid metabolism (16). VEGF-B is related to the promotion of lipid accumulation and lipotoxicity in podocyte. VEGF-C can disrupt the glomerular filtration barrier (17). Vascular endothelial growth factor C is involved in angiogenesis and lymphogenesis. Some research showed that amelioration of intrarenal inflammation and fibrosis is associated with attenuated lymphatic proliferation in the kidney (18).

The VEGF signaling pathway is one of the most important pathways involved in podocyte-endothelial cell interaction. In the early stage of DN, the VEGF signaling pathway is activated in the glomerulus, which leads to cell dysfunction and abnormal angiogenesis, eventually promoting glomerular cell hypertrophy and albuminuria (19).

In the kidney, VEGF-A is mainly expressed on glomerular podocytes. VEGF R2 on the surface of endothelial cells is phosphorylated after binding with VEGF and increases intracellular calcium ion level through PI3K/Akt signaling pathway. After binding with calmodulin enhances eNOS activity and increases NO production (20–22). On the pathological conditions of hyperglycemia, excessive production of VEGF-A produced by podocytes can induce abnormal angiogenesis of endothelial cells, leading to immature capillaries in the glomerulus (14). Overactivation of VEGFR2 also increases the production of ROS, especially superoxide (O_2_-), aggravating endothelial injury, and leading to the occurrence and progression of the renal microvascular disease (23, 24). Increased expression levels of VEGF-A in the blood lead to changes in GBM and a decrease in the glycocalyx of endothelial cells (25), which further promotes the increase of vascular permeability and causes leakage. Although the activation of the VEGF/VEGFR signaling pathway in the early stage of DN leads to the formation of new blood vessels and glomerular injury, excessive loss of podocytes in the late stage leads to the weakening of VEGF signaling, which further leads to vascular thinning and renal fibrosis, aggravating the development of DN (26). Both loss and overexpression of VEGF lead to glomerular abnormality; loss of VEGF-A prevents glomerular angiogenesis and the development of a glomerular filtration barrier (27). Overexpression of VEGF-A in podocytes is similar in different renal diseases (28). For example, there is no difference between glomerulopathy caused by overexpression of VEGF164 and glomerulopathy caused by early diabetes nephropathy (15, 29).

Nowadays, increased use of glucose-lowering agents and glycemic control have not resulted in a reduced prevalence of DN. More and more pathogeneses are considered to drive the development of DN (30). Thus, we looked for new mechanisms. In muscle, VEGF-B has been shown to control lipid accumulation through regulation of endothelial fatty acid (FA) transcytosis, and it may thus be a potential target in treating type 2 diabetes (T2DM) (31) VEGF-B was overexpressed in podocytes, while VEGFR1 was mainly found to be expressed on endothelial cells. The article showed reducing VEGF-B signaling could ameliorate glomerular lipotoxicity and, as a consequence, the progression of DN. Glomerular VEGF-B levels were upregulated in subjects with DN, suggesting that anti-VEGF-B treatment may be useful as a therapeutic strategy to treat DN in humans (32).

VEGF-C can protect endothelial cells from the influence of VEGF-A reduction on cell permeability and plays a role in protecting endothelial cells (33). Additionally, apart from blood glucose, increased levels of advanced glycation end products (AGEs) can also promote VEGF expression in podocytes, thereby increasing the oxidative stress response of endothelial cells and podocytes (34, 35).

#### 3.1.2 Transforming growth factor

TGF-β is a multifunctional dipeptide. It is a family of factors that promote cell growth and differentiation, consisting of more than 30 proteins with similar structures (36). TGF-β1 is one of the fibrogenic factors secreted by glomerular endothelial cells, glomerular mesangial cells, and renal tubule epithelial cells.

Unlike the VEGF signaling pathway necessary to maintain the glomerular filtration barrier, in a high glucose environment, the over-activation of the TGF- β signaling pathway is harmful to mesangial cells, podocytes, and endothelial cells. TGF- β 1 can induce the occurrence of epithelial-mesenchymal transition (EMT) (37) and the accumulation of extracellular matrix (ECM) in renal tubular epithelial cells, which is closely related to the progression of renal interstitial fibrosis in DN. Inhibiting the TGF- β 1 signaling pathway can reduce EMT and fibrosis of DN (38).

In addition, in the diabetes mouse model, TGF- β signaling pathway can induce dedifferentiation or apoptosis of endothelial cells and podocytes (39). TGF-β1 secreted by endothelial cells binds to TGF-βR1 of podocytes, which can activate downstream Smad signals, including Smad2, Smad3, and Smad7 (40). Activation of Smad2/3 promotes the secretion of ECM, leading to glomerular sclerosis and fibrosis, while Smad7 induces apoptosis of podocytes by blocking the activity of NF-kB. Meanwhile, Smad7 can directly activate caspase3. Cysteingl aspartate specific protease(Caspase)is an important cytokine in the molecular mechanism of cell apoptosis. Smad7 and p38MAPK induce podocyte apoptosis by activating caspase3 (41). Similarly, in high glucose condition, the endothelial cell secretes TGF- β to promote apoptosis and ultimately aggravates renal microvascular disease (42).

#### 3.1.3 Angiotensin/angiopoietin receptor 2

Angiotensin (Ang 1/Ang 2) plays an important role in maintaining endothelial integrity and participates in the pathophysiological process of DN. Ang-1 is mainly produced by podocytes and can promote angiogenesis and microvascular growth by binding to the Tie2 receptor expressed by glomerular endothelial cells, reducing the permeability of endothelial cells and regulating the VEGF signaling (43, 44).

Ang2 is mainly produced by GECs (Glomerular Endothelial Cells) and inhibits Ang1 through competitive binding to the Tie-2 receptor (45). Studies have demonstrated that Ang-2 expression is up-regulated in the glomerulus of diabetic nephropathy patients, which antagonists Ang-1 induced Tie 2 activation, and thus inhibits the anti-apoptotic effect of Ang-1 on endothelial cells (46). In pathological conditions of DN, decreased glomerular VEGF-A expression is accompanied by increased Ang-2/Ang-1 ratio, resulting in increased apoptosis of endothelial cells (47).

#### 3.1.4 Endothelin

Endothelin (ET-1) is a vasoconstrictive peptide mainly produced by GECs.ET-1 binds to two subtypes of receptors, the endothelin A receptor (ETAR) and the endothelin B receptor (ETBR) (48). Studies about animal models of diabetes have shown the expression of ET-1 in glomeruli was increased by 5 times compared with the receptor, suggesting that ET-1 plays an important role in DN (49). In the pathological conditions of diabetes, ET-1 is associated with vasoconstriction, kidney injury, mesangial hyperplasia, glomerulosclerosis, fibrosis, and inflammation (50). ETAR is mainly expressed in podocytes of the glomerulus. ET-1 combined with ETAR to promote vasoconstriction, cell proliferation, fibrosis, podocyte injury, and inflammatory response. ETBR is mainly distributed in glomerular endothelial cells and renal tubular epithelial cells. ET-1 combined with ETBR can promote vascular dilation, anti-proliferation, and anti-fibrosis, and play a protective role.

ET-1 can also be secreted by podocytes. In DN progression, TGF-β signaling is activated in podocytes, followed by increased secretion of ET-1. Binding to ETAR in GECs mediates mitochondrial oxidative stress and adjacent endothelial cell dysfunction (35, 51, 52). Selective blocking of ETAR has been demonstrated to reduce the expression of chemokines and cytokines, as well as decrease the secretion of various mediators of renal fibrosis. It can prevent podocyte loss, albuminuria, and glomerulosclerosis (53).

ET-1 seems to be a key mediator in podocytes-to-ECs and ECs-to-podocytes communications promoting cell injury in several renal pathologies including DN. Endothelin A (ETA) receptor antagonist—Atrasentan entered the field of vision as a new drug, and has got some achievements in DN. The SONAR (The Study of Diabetic Nephropathy with Atrasentan) (54) is the first trial to evaluate the long-term effectiveness of ETA receptor antagonists on albuminuria DN. The results showed that Atrasentan reduced the risk of progression to CKD (including DN) by 35% compared with placebo and was safe and well tolerated.

### 3.2 Endothelial cell and mesangial cell

GECs directly contact and interact with mesangial cells in structure. In the high glycemic environment, the crosstalks between GEC and GMC (Glomerulus Mesangial Cells) induce the inflammatory responses of the kidney and glomerulosclerosis (55, 56), which damage the integrity of the glomerular tissue and cause renal dysfunction.

#### 3.2.1 Platelet-derived growth factor

PDGFB is mainly expressed in GECs, while PDGFR-β is mainly expressed in MCs. PDGFB/PDGFR is the main medium in the crosstalk between GEC and MCs (57). During glomerular development, the interaction of GEC and MCs promotes mesangial cell maturation and maintains through the PDGF-B/PDGFR signaling pathway (58). The PDGFB/PDGFR signaling pathway is activated in the glomeruli of diabetic mice, which promotes the progression of DN, and the mechanism may be related to oxidative stress and mesangial expansion (59). It has been demonstrated that GECs paracrine PDGFB to regulate mesangial cell proliferation, and the inhibition of PDGFB and its receptor can inhibit mesangial proliferation in diabetic rats (51). The expression of PDGFB in the kidney of diabetic nephropathy patients is significantly increased (60). The non-expression of PDGFB and its receptor PDGFR-β can reduce the accumulation of extracellular matrix and mesangial cells proliferation (61). In addition to mesangial cells, renal vascular smooth muscle cells (SMC) are also the target of PDGF-B signaling, which accelerates the development or neovasculature of blood vessels by promoting the proliferation of SMC (62).

#### 3.2.2 Endothelin-1

ET-1 may be involved in the occurrence of diabetic vascular diseases through mesangial cell proliferation, promoting fibrosis and inflammation.ET-1, one of the most effective vasoconstrictors and a growth factor for mesangial cells (48, 63), is positively associated with increased albumin excretion in patients with diabetic nephropathy. ET-1 is secreted by endothelial cells, binds to ETAR and ETBR on mesangial cells, and exerts its effect. For example, mesangial proliferation and ECM accumulation are accelerated by up-regulating the expression of ECM-related genes (64). Some studies have shown that HG activates RhoA/ROCK signaling pathway in mesangial cells and promotes the progression of DN. ET-1 mediates ETAR activation in mesangial cells and enhances the expression of ECM-related genes (65, 66). Studies have shown that high glucose activates the RhoA/ROCK pathway in mesangial cells and promotes the progression of DN, which also depends on the secretion of ET-1. On the other hand, the combination of ET-1 with ETBR can inhibit the NF-κB signaling pathway and reduce the secretion of ET-1 by endothelial cells. This negative feedback reduces the inflammatory responses of endothelial cells (63).

### 3.3 Endothelial cell and renal tubular epithelial cell

In the early stage of DN, renal tubules are damaged to varying degrees, and tubular epithelial cell damage can lead to abnormal endothelial cell function. The crosstalk between the two kinds of cells induces inflammatory responses and promotes the occurrence and progress of DN.

#### 3.3.1 Inflammation

As the first barrier of the glomerular filtration membrane, GECs come into directly contact with substances in the circulating blood and are more vulnerable to damage by inflammatory factors (67). Except for functioning in the interaction between podocytes and endothelial cells, VEGF/VEGFR signaling pathway also plays an important role in the interaction between epithelial cells and endothelial cells in the renal tubulointerstitial. In the diabetic environment, tubule epithelial cells (TECs) are susceptible to hemodynamic changes in metabolic disorders, resulting in the secretion of multiple inflammatory mediators, leading to interstitial inflammation. Urinary albumin in DN patients activates TECs to produce pro-inflammatory factors such as CRP, IL, TNF-α, NF-κB, and ROS, which can lead to GEC injury, apoptosis, and EndMT. Stimulated by inflammatory factors, the glomerular vascular network suffered apoptosis and necrosis, and the structure and function of GECs were destroyed. The injured GEC reduced the blood supply to the renal tubules, resulting in increased TEC injury (68). The interaction aggravates the progress of DN.

#### 3.3.2 Vascular endothelial growth factor

In addition to playing a role in the interaction between podocytes and endothelial cells, VEGF/VEGFR signaling also plays an important role in the interaction between epithelial cells and endothelial cells in the renal tubulointerstitial. In the renal tubulointerstitial, VEGF-A can also be synthesized and secreted by renal tubule epithelial cells (TECs) and subsequently combines with VEGFR of endothelial cells (GECs) to regulate the structure and function of GECs (69). VEGF/VEGFR activation in early DN leads to neovascularization. However, in the late stage of DN, the aggravation of TEC injury and deletion in late DN is accompanied by decreased production of VEGF-A, decreased endothelial cell angiogenesis and permeability of endothelial cells, and endothelial function destruction (70). Subsequently, eNOS deficiency, NO reduction, interstitial ischemia, and hypoxia occur, leading to TEC damage, decreasing VEGF-A synthesis, and aggravating a vicious cycle in disease progression (71).

### 3.4 Podocyte and glomerular mesangial cell

The abnormalities of podocytes and mesangial cells play a key role in the development of DN. Recent researches suggest that crosstalks between the two types of cells may be mediated by exosomes. Exosomes are spherical extracellular vesicles (ECVs) with a phospholipid bilayer membrane structure that are 40-100 nm in diameter and contain a variety of molecules, such as proteins, lipids, DNA, miRNA, and LncRNA. Normally, exosomes can be secreted by kidney cells, such as podocytes and mesangial cells. In recent years, a large number of data have proved that exosomes are involved in the glomerular, renal tubule, and tubulointerstitial lesions, which are related to the pathological changes and prognosis of DN (72, 73). In the high glucose condition, exosomes released by GMCs may affect podocyte function by carrying TGF-β1, participating in the pathological process of DN, and eventually leading to albuminuria (74, 75).

Podocyte and Endothelial cell:In the pathological condition of high glucose, the abnormal renal expression of VEGF, angiopoietins, TGF- β, and endothelin-1 in early Diabetic nephropathy (DN) induces endothelial cell dysfunction and contributes to the disappearance of podocyte foot processes.

Endothelial cell and Mesanginal cell:PDGFB secreted by GECs binds to PDGFR-βR on mesangial cells, which contributes to the development of mesangial cells. ET-1 is a growth factor of mesangial cells. ET-1 may be involved in the pathogenesis of diabetic vascular diseases through mesangial cell proliferation, promoting fibrosis and promoting inflammation.

Podocyte and Macrophage:Macrophage is the main immune cell that causes kidney injury in DN. Infiltration, recruitment, and activation of macrophages can lead to the generation and release of many inflammatory factors, pro-fibrotic factors, and anti-angiogenic factors, such as TNF-α, ROS, IL-1, IL-6, TGF-β, and VEGF, which can interact with podocytes.

### 3.5 Macrophage and renal tubular epithelial cell: extracellular vesicles

The single-cell sequencing study by Fu et al. provided direct evidence that macrophages were the main infiltrating immune cells in the kidney tissue of diabetic mice, and M1 macrophages were more significant in early DN (76). Experimental evidence also confirmed that there were different degrees of immune cell infiltration in renal tissues of DN patients, which was related to DN staging (77). All these phenomena suggest that in the diabetic environment, macrophages interact with cytokines, causing renal function damage and accelerating the progression of DN (78).

#### 3.5.1 Toll-like receptor

Toll-like receptor (TLRs) protein is an important receptor that can regulate immune response and inflammatory diseases. TLR4 expression was increased in macrophages of diabetic nephropathy patients, while TLR2 expression was not changed (79). The expression level of TLR4 was directly related to the infiltration of tubulointerstitial macrophages and was inversely proportional to the glomerular filtration rate. In the TLR4 knockout diabetes mice, the infiltration of interstitial macrophages, proteinuria, and progress of renal function was significantly improved. Itsmechanism was confirmed by cell experiments: high glucose induces TLR4 expression by activating PKC in human proximal tubule epithelial cells, and the activation of IkB/NF-κB leads to up-regulated expression of IL-6 and CCL-2 and leads to the inflammatory response (79). Therefore, TLR4 mediates the action of macrophages and renal tubular epithelial cells to increase the tubulointerstitial inflammatory response and promote diabetic kidney injury.

#### 3.5.2 Exosome

TEC-derived exosome (EVe) activates the inflammatory phenotype of macrophages, inducing the expression and release of proinflammatory cytokines, and inducing the release of macrophage-derived exosome (EVm). EVm can also induce the apoptosis of lipotoxic TECs, thus forming a vicious cycle, and promoting kidney inflammation and damage in DN (80). Mechanism studies have shown that Eve contains high levels of miR-19b-3p. It can activate the NF-κB signaling pathway by targeting the inhibition of SOCS-1, leading to the phenotype polarization of M1 macrophages, further increasing the expression of MCP-1, IL-1β, and other renal inflammatory cytokines, and promoting the occurrence of renal tubulointerstitial inflammation in diabetic nephropathy (81). Vascular lesions caused by diabetes lead to renal ischemia and hypoxia. Li et al. confirmed that renal tubular epithelial cells in an anoxic environment could release mirNA-23A-rich exosomes, which could be absorbed by macrophages and transformed into pro-inflammatory phenotypes through the HIF-1α pathway, promoting tubulointerstitial inflammation (82). These studies confirmed that microRNAs in exosomes mediate interactions between renal tubular epithelial cells and macrophages in DN tubulointerstitial inflammation.

#### 3.5.3 Macrophage and podocyte

Infiltration, recruitment and activation of macrophages can lead to the generation and release of many inflammatory, pro-fibrotic and anti-angiogenic factors. For example, TNF-α, ROS, IL-1, IL-6, TGF-β and VEGF can interact with podocytes (83).In addition, cytokines act on podocytes, the major components of the filtration barrier, through multiple signaling pathways, such as p38 MAPK, NF-κB, Toll-like receptors, or proteins, ultimately damage renal cell and aggravate the progression of the disease.

### 3.6 Pericyte and other cells

Pericytes are a class of undifferentiated cells. It is generally believed that mesenchymal cells adjacent to microvascular endothelial cells are pericytes (84). In the kidney, pericytes are highly specialized cells that make up 30% of the total tissue and are involved in the regulation of glomerular ultrafiltration. It plays an important role in the pathophysiological activities of microvascular. PDGFR-α and -β were expressed only in pericytes and myofibroblasts. Inhibition of PDGF signaling by imatinib or neutralizing PDGFR antibodies can reduce macrophage infiltration and fibrosis (85). Hu et al. showed that C1q tumor necrosis factor-associated protein-3 could reduce mesangial cell proliferation and extracellular matrix accumulation induced by high glucose, and inhibit pericytes to differentiate into mesangial cells (86). This evidence suggests that the interaction between pericytes and other cells also plays a significant role in the progression of DN.

Endothelial cell and Renal tubular epithelial cell:The crosstalk between endothelial cells and renal tubular epithelial cell plays an important role in the occurrence and development of DN. There are many signaling pathways between GECs and TECs, in which crosstalk plays a vast role. The abnormal secretion of VEGF and inflammatory factors(such as CRP、IL-6、TNF-αand so on) promote injury to GECs during the progress of DN.

Tubular epithelial cells and macrophages:Macrophage infiltration around renal tubular epithelial cells (TECs) is a hallmark of DN. In the diabetic environment, macrophages interact with tubule epithelial cells through cytokines, causing renal function damage and accelerating the progression of DN.

Pericyte and other cells:There are many common signaling pathways among podocytes, mesangial cell endothelial cells, and pericytes, in which crosstalk plays a vast role, Such as C1q(Complement 1q).

### 3.7 New therapeutic perspectives

Recently, new therapeutics including sodium-glucose transport protein 2 (SGLT2) inhibitors, endothelin antagonists, glucagon like peptide-1 (GLP-1) agonists, and mineralocorticoid receptor antagonists (MRA), have provided additional treatment options for patients with DN.

Recently, SGLT2 inhibitors have emerged as a new class of drugs, to control blood sugar by increasing the excretion of sugar in the urine. Ipragliflozin, was injected into streptozotocin induced diabetic mice to reduce the blood glucose level of diabetic mice. The experimental results showed that it alleviated the damage of diabetic endothelial function, improved the phosphorylation of eNOS, and reduced the expression of inflammatory molecules (87).

Furthermore, Glucagon-like peptide-1 (GLP-1) is an intestinal hormone. It has the properties of increasing insulin secretion and inhibiting glucagon secretion after eating. It plays an important role in maintaining blood glucose homeostasis (88). The inhibition of oxidative stress, inflammation, fibrosis, and induction of natriuresis have been mainly implicated as mechanisms underlying the attenuation of DN by GLP-1 receptor agonists.GLP-1 receptor agonists have a renal protective effect in addition to hypoglycemic effects (89).

Dipeptidyl peptidase-4 (DPP-4) inhibitors reduce the levels of blood glucose by increasing the half-life of short-lived endogenous incretins, such as GLP-1 and glucose-dependent insulinotropic polypeptide (90). DPP-4inhibitors (insulin-based therapy), a new class of hypoglycemic agents for clinical practice, their role in diabetic nephropathy, with a particular focus on renal protection and alternative markers of cardiovascular disease (91). Trials such as SAVOR-TIMI 53 (92) and CARMELINA (93) showed a possible reduction in albuminuria and improvement in the histological changes of kidney in patients receiving DPP-4 inhibitors.

In addition to GLP-1 and SGLT2 discussed earlier, there is a new class of drugs called MRAs (Mineralocorticoid receptor antagonists).MRAs are drugs that inhibit the effect of aldosterone on mineral mineralocorticoid receptor (MR), such as fenelidone (94). MRAs block MR-mediated sodium reabsorption and MR Overactivation in renal tissues. In patients with diabetic nephropathy, MRA reduces the upregulation of pro-inflammatory mediators, including TGF-β, PDGF,CCL2 and so on (95). In preclinical studies, the protective effect of MRAs in animal models of diabetes nephropathy was reported in 2001 (96). Mineralocorticoid receptor-mediated inflammation has been proposed to be partially caused by injury, oxidative stress or cell apoptosis (95).

## 4 Discussion

**Figure 1:**
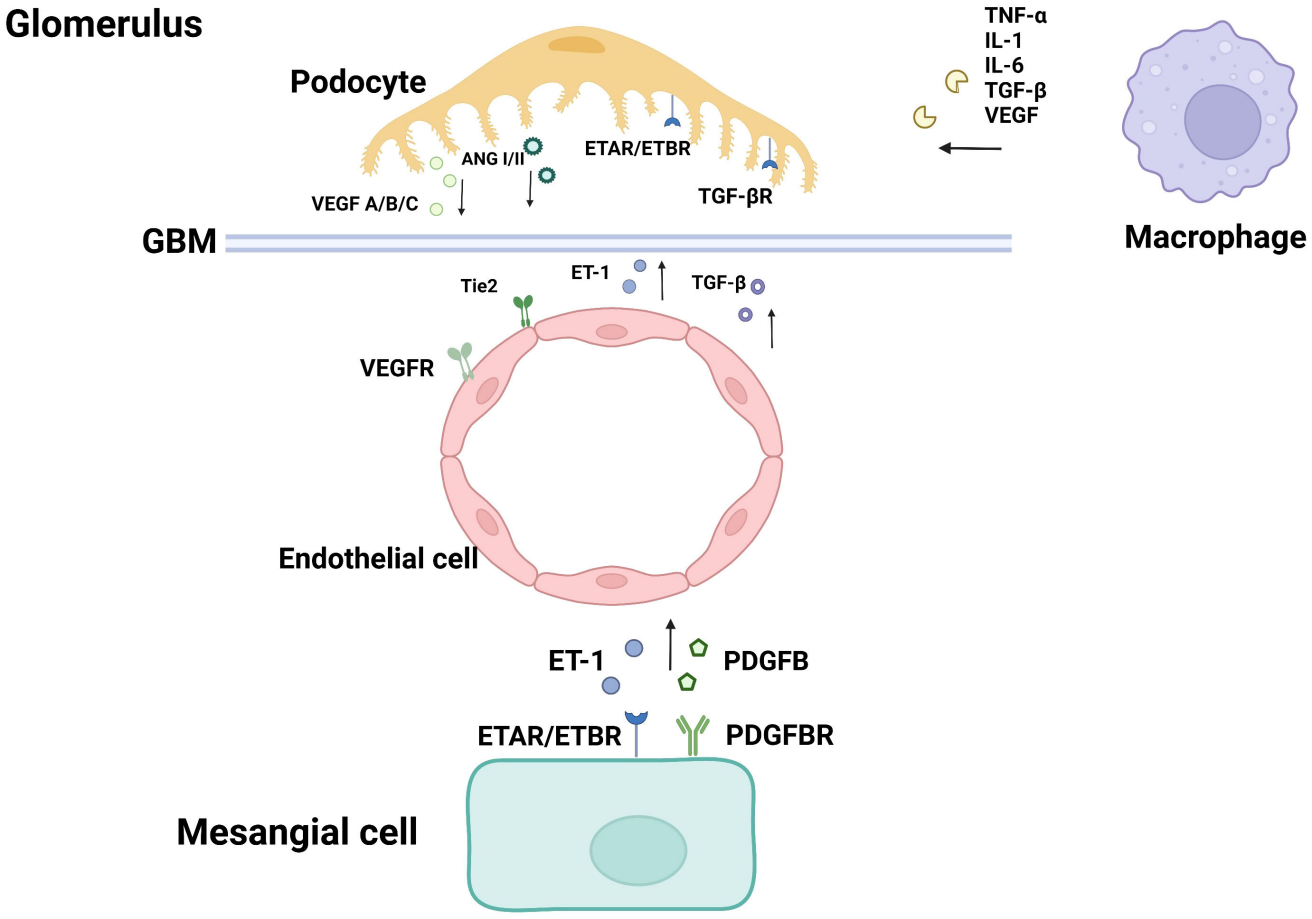
Cellular crosstalk in the glomerulus.

**Figure 2:**
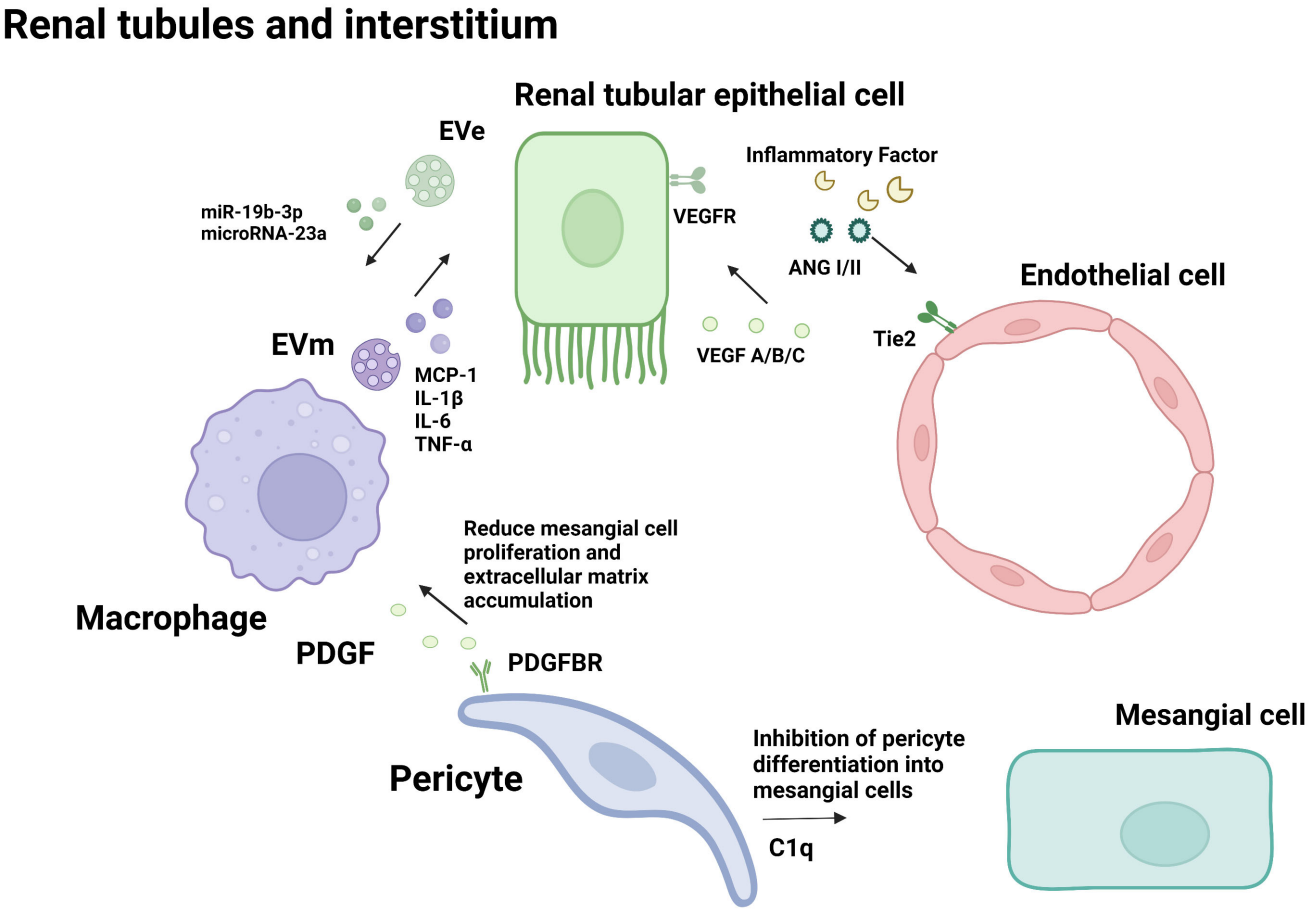
Cellular crosstalk in the renal tubules and interstitium.

Glomerular endothelial cells, podocytes and renal tubular epithelial cells play important roles in the pathophysiological progression of DN. Many studies have proved that crosstalk among them, as well as neighboring pericytes and immune cells, which is related to the progression of diabetic nephropathy (As shown in the **Figures 1**, **2**). The cell damage caused by this crosstalk is oxidative stress; macrophage infiltration, inflammatory response, the increasing secretion of TGF-b; abnormal angiogenesis caused by increased secretion of VEGF. There is a table (As shown in the **Table 1**) with important cellular crosstalks that contribute to understand the progression of diabetic nephropathy.

**Table 1:** The full search words about crosstalk between renal cells and other cells in the DN.

Cell	Cytokine/Receptor
Endothelial cell and podocyte	Vascular endothelial growth factor-A、B、C/Receptor
	Transforming growth factor -β/Receptor
	Angiotensin I/II、Tie2 receptor
	Endothelin-1、Endothelin A/B receptor
Endothelial cell and mesangial cell	Platelet-derived growth factor-B/Receptor
	Endothelin-1、Endothelin A/B receptor
Endothelial cell and renal tubular epithelial cell	Inflammation factor
	Vascular endothelial growth factor、Vascular endothelial growth factor receptor
Podocyte and glomerular mesangial cell	Exosome
Macrophage and renal tubular epithelial cell:	Toll-like receptor 2/4
	Exosome
Macrophage and podocyte	inflammatory factor, pro-fibrotic factor, anti-angiogenic factor
Pericyte and other cells	Platelet-derived growth factor

At present, many studies need to be further clarified on the role of cell crosstalk in the occurrence and development of DN. For example, the mechanism of VEGF and TGF-β. The VEGF-A and VEGF-C crosstalks are controversial. VEGF-A is a critical mediator of angiogenesis and vasculogenesis and is involved in the formation of glomerular filtration barriers. Human kidney biopsies showed high VEGF-A expression at early stages of DN, and lower VEGF-A expression in patients with more advanced stage of DN because loss of podocyte (14). Studies suggest that in the diabetic nephropathy, the disruption of podocyte function after VEGF-A depletion arises from endothelial cell dysfunction. Excessive VEGF-A production results in neovascularization leading to pathologic microangiopathy. In the later stages, the reduction of VEGF signaling may occur due to the loss of podocytes, contributing to vascular rarefication and renal fibrosis. VEGF-C is mainly involved in lymphangiogenesis and binds to VEGFR-2, VEGFR-3.Some studies showed that renal inflammation and fibrosis were improved in diabetic mice by down-regulating VEGF-C and VEGFR-3 expression (97). However, in DN, a chronic proinflammatory state, the overgrown lymph eventually becomes incomplete and dysfunctional due to chronic upregulation of VEGF-C (98). Its detailed and systematic mechanisms still need further study.

In addition, in DN, early studies have shown that that crosstalk between TGF-β and hormones has a complex mechanism in DN. The data on diabetic nephropathy and TGF signaling pathway appear to be controversial. The results suggest that sex, sex hormones and diabetic conditions influence differences in expression of TGF-β1, its receptor and bone morphogenetic protein 7 (BMP7). Complex crosstalk between sex hormones, sex-dependent expression pattern and profibrotic signals for the precise course of DN development (92). So, we still have a lot to figure out about crosstalk between cells.

The interaction of glomerular endothelial cells, podocytes, renal tubular epithelial cells, and other cells is closely related to the progression of DN. The pathogenesis of DN is numerous and complex, and it remains unknown. The interaction among cells is the key factor that promotes DN progression. The new treatment plan to improve the damage to kidney cells and maintain the normal crosstalk among cells may become a new strategy for the prevention and treatment of DN in the future. Targeted drug research on crosstalk among cells to maintain cell function may be a new perspective, which will provide a brand-new strategy for the prevention and treatment of DN.

## Author contributions

KZ, YZ, and ZF oversaw the preparation of the manuscript and wrote the first draft. XC, GC, and QH design this topic and give guidance. All authors contributed to the article and approved the submitted version.
